# Herbal monomer–based therapeutic insights from traditional Chinese medicine in myocardial infarction

**DOI:** 10.3389/fmolb.2025.1721931

**Published:** 2026-01-07

**Authors:** Nan Bian, Libo Xia, Xianqiu Xiong, Yingyu Chen, Ying Chen

**Affiliations:** 1 Changchun University of Chinese Medicine, Changchun, Jilin, China; 2 The First Affiliated Hospital to Changchun University of Chinese Medicine, Changchun, Jilin, China; 3 Guizhou Institute of Precision Medicine, The Affiliated Hospital of Guizhou Medical University, Guiyang, China; 4 School of Medical Information and Engineering, Guangdong Pharmaceutical University, Guangzhou, China

**Keywords:** myocardial infarction, Quercetin, Mendelian randomization, traditional Chinese medicine, single-cell RNA-sequence

## Abstract

Myocardial infarction (MI) is a leading cause of morbidity and mortality globally, often resulting in heart failure due to adverse cardiac remodeling triggered by inflammation and fibrosis. Traditional Chinese Medicine (TCM), particularly compounds like Quercetin from Licorice and Peony, has shown promise in modulating inflammation and oxidative stress in cardiovascular diseases. This study integrates bioinformatics and experimental validation to explore the therapeutic potential of Quercetin in MI. Using Mendelian Randomization (MR) and colocalization analysis, we identified key MI-related genes, such as VEGFA, PTK2, and GGT1, whose expression is influenced by Quercetin. Bioinformatics tools predicted these genes as targets of Quercetin, with molecular docking revealing stable interactions between the compound and these genes. Single-cell RNA-sequencing of MI samples confirmed the expression of these genes in cardiac muscle cells (CMs) and macrophages, highlighting their role in tissue repair and inflammation. In experimental models, Quercetin treatment significantly altered the expression of these genes, enhancing myocardial cell recovery and reducing infarct size. This study provides molecular insights into how Quercetin and other TCM compounds could modulate critical pathways involved in MI recovery, supporting their potential as adjunct therapies. The findings bridge traditional medicine with modern bioinformatics, opening new avenues for therapeutic strategies to improve cardiac function and patient outcomes in MI.

## Introduction

Myocardial infarction (MI) is a severe cardiovascular condition triggered by inadequate blood supply to the heart due to blockages in coronary arteries, resulting in ischemia and myocardial tissue necrosis (Dauerman and Ibanez, 2021; Jacoby and Nesto, 1992). As a major cause of morbidity and mortality globally, MI often leads to long-term complications such as heart failure (HF), which significantly impacts patient survival and quality of life. While conventional treatments, including percutaneous coronary intervention (PCI) and coronary artery bypass grafting (CABG), improve prognosis, post-MI heart remodeling remains a critical issue. Notably, the acute inflammatory response and chronic low-grade inflammation significantly contribute to the progression of adverse ventricular remodeling and HF following MI. The process involves macrophage activation, extracellular matrix changes, and pro-inflammatory cytokine release, leading to further myocardial injury (Chapman et al., 2025). The imbalance between pro-inflammatory and anti-inflammatory signals in the early and late phases of MI-induced cardiac remodeling contributes significantly to heart failure.

Traditional Chinese Medicine (TCM), long revered for its efficacy in treating a variety of diseases, including cardiovascular conditions, offers promising therapeutic options (Jiang et al., 2022; Lu et al., 2015). Licorice (Glycyrrhiza uralensis), Peony (Paeonia lactiflora), and Quercetin, an active flavonoid, have been studied for their cardioprotective properties. Licorice, with its well-established anti-inflammatory and antioxidant effects, has been found to alleviate the systemic inflammatory response after MI and improve myocardial function. Peony, known for its ability to modulate the immune system and reduce oxidative stress, plays a role in reducing heart tissue damage and inflammation (Dong-mei et al., 2013; Zhang et al., 2019; Liu et al., 2011). Quercetin, a potent flavonoid, is widely recognized for its antioxidative and anti-inflammatory properties, which may reduce infarct size, improve vascular function, and reduce myocardial fibrosis (Hu et al., 2024). These compounds, used in TCM formulations, could offer a complementary approach to modern therapies by modulating the immune-inflammatory response and promoting tissue repair after MI. The current clinical use of Quercetin has gained significant attention due to its potential to modulate cardiac function post-MI (Liu et al., 2024). Several studies have demonstrated that Quercetin can inhibit oxidative stress and inflammation, reduce myocardial apoptosis, and promote repair after ischemic injury. Notably, recent research suggests that Quercetin might influence macrophage polarization, particularly the shift towards the M2 macrophage phenotype, which is crucial for tissue repair and fibrosis resolution. Furthermore, Quercetin’s interaction with signaling pathways like NF-κB and Nrf2 further supports its cardioprotective effects (Kozhukhov et al., 2024; Rao et al., 2024).

Despite the promise of these compounds, the precise molecular mechanisms through which they exert their therapeutic effects remain inadequately understood. The identification of specific molecular targets is critical for optimizing TCM formulations and integrating them into mainstream therapeutic strategies. This is where modern bioinformatics tools, including Mendelian Randomization (MR) and colocalization analysis, can offer profound insights into the genetic underpinnings of these therapies. By integrating GWAS (Genome-Wide Association Studies) data and expression quantitative trait loci (eQTL) signals, we can identify potential target genes that mediate the effects of Quercetin in MI treatment. Additionally, through bioinformatics, we can explore the interaction between these compounds and key proteins involved in inflammation, fibrosis, and myocardial repair, leading to the discovery of novel therapeutic targets.

This study aims to bridge traditional Chinese medicine with modern genetic tools to investigate the molecular mechanisms of Quercetin and other TCM components in treating MI. We will employ Mendelian Randomization (MR) to assess the causal relationships between identified genetic variants, colocalization analysis to pinpoint shared causal variants between eQTLs and GWAS signals, and bioinformatics methods to analyze gene-environment interactions and predict drug-target interactions. These approaches will provide deeper insights into the potential molecular targets of Quercetin, paving the way for more targeted and effective treatments for MI.

## Methods

### Collection of active ingredients and potential targets

For licorice, active compounds and their potential targets were identified using the Traditional Chinese Medicine Systems Pharmacology Database and Analysis Platform (TCMSP). Compounds were screened based on the criteria of oral bioavailability (OB ≥ 30) and drug-likeness (DL ≥ 0.18). The protein names of the identified targets were converted using the UniProt database ([https://www.uniprot.org/] (https://www.uniprot.org/)).

For peony, the TCMID database was used to screen for active compounds, which were then further evaluated using the Swiss ADME software ([http://www.swissadme.ch/] (http://www.swissadme.ch/)) based on pharmacokinetic properties such as absorption, distribution, metabolism, and excretion (ADME). Screening criteria included high gastrointestinal absorption (GI absorption: high), and meeting at least three out of the following Lipinski, Ghose, Veber, Egan, and Megge rules (yes). After identifying suitable compounds, target prediction was carried out using the SwissTargetPrediction database ([http://www.swisstargetprediction.ch] (http://www.swisstargetprediction.ch)).

### Construction of component-target network

The component-target data were imported into Cytoscape 3.10.0 software, where the NetworkAnalyzer tool was used to compute the topological properties of the network. The resulting “component-target” network was adjusted to visualize the regulatory relationships between components and targets.

### GO and KEGG enrichment analysis

The drug-related targets were subjected to Gene Ontology (GO) and Kyoto Encyclopedia of Genes and Genomes (KEGG) pathway enrichment analysis using the DAVID tool ([https://davidbioinformatics.nih.gov/] (https://davidbioinformatics.nih.gov/)). For GO enrichment, P-values were used as the selection criterion, and the top 10 biological terms with the lowest P-values were selected to generate a bar chart. For KEGG pathway enrichment, the same P-value threshold was applied, and the top 30 most significantly enriched signaling pathways were visualized using a bubble plot for further analysis.

### Druggable targets of peony and licorice compounds

Building on the target genes identified for the active constituents of Paeonia (peony) and Glycyrrhiza (licorice), we intersected these targets with the curated list of druggable genes reported in the study “The druggable genome and support for target identification and validation in drug development.” The overlap was designated as the set of druggable TCM targets for peony and licorice.

### Data source: eQTLGen consortium database

In this study, exposure data was obtained from the eQTLGen consortium database, which aims to explore the genetic architecture of blood gene expression and investigate the genetic basis of complex traits. The eQTLGen project is currently in its second phase, focusing on large-scale, genome-wide meta-analyses of blood gene expression. This resource provides valuable insights into the genetic factors influencing gene expression in blood, helping to identify potential genetic variants associated with various traits and diseases.

Outcome data were sourced from the FinnGen database (finn-b-I9\_MI\_EXNONE), a large-scale genomic research project that integrates genetic data and health records from the Finnish population to investigate the relationship between genes and diseases. The FinnGen project combines biological samples and electronic health records from Finland, covering a broad range of diseases and phenotypes, and providing extensive information on genetic variations. This resource is not only essential for basic research but also holds significant potential for personalized medicine and disease prevention, fostering a deeper understanding of complex diseases and contributing to the development of therapeutic strategies. Specifically, the dataset used for this analysis includes 12,801 cases of myocardial infarction and 205,991 controls.

### Mendelian randomization and colocalization analysis

Based on the intersecting genes identified from peony and licorice active compound targets and the druggable gene set, we conducted Mendelian randomization (MR) analysis to assess potential causal relationships with disease outcomes. Colocalization analysis was further performed using the Coloc R package, a Bayesian framework designed to evaluate whether two genetic association signals—such as expression quantitative trait loci (eQTLs) and genome-wide association study (GWAS) signals—share a common causal variant within the same genomic locus. Posterior probabilities were calculated under five competing hypotheses, allowing us to quantify the likelihood that eQTL and GWAS associations colocalize. This approach provides statistical evidence for shared genetic mechanisms, thereby enhancing the understanding of molecular pathways and regulatory networks underlying complex diseases.

### Single-cell RNA sequencing data processing and analysis

Publicly available single-cell RNA sequencing (scRNA-seq) data of myocardial infarction (MI) samples were obtained from the Gene Expression Omnibus (GEO) database under accession number GSE214611. The dataset contains myocardial tissue samples collected from MI models, which were processed and sequenced using the 10× Genomics Chromium platform.

The raw gene-barcode matrices were downloaded and analyzed using Seurat (version 4.1.0) in R. Low-quality cells with <200 detected genes or >10% mitochondrial gene content were excluded. The data were normalized and log-transformed, followed by principal component analysis (PCA) and uniform manifold approximation and projection (UMAP) for dimensionality reduction. Clustering was performed using the FindNeighbors and FindClusters functions with a resolution of 0.5. Cell types were annotated based on the expression of canonical marker genes. Cardiomyocytes (CMs) were identified by the expression of TNNT2 and ACTC1; endothelial cells were marked by PECAM1 and VWF; fibroblasts expressed COL1A1 and DCN; macrophages were defined by CD68 and CD163; smooth muscle cells (SMCs) were characterized by ACTA2 and MYH11; cycling cells showed high expression of MKI67 and TOP2A; and neuronal cells were distinguished by NEFL and SNAP25.

We calculated module gene scores for key gene sets using the AUCell package to assess activity levels across cell populations. The module scoring results were visualized using UMAP and violin plots. As shown in Figure 4E, cells were divided into seven main subtypes (CM, cycling, endothelial, fibroblast, macrophage, neuronal, and smooth muscle cells). Consistent with our analysis of the heart failure dataset, the high-scoring groups in the MI dataset exhibited a higher proportion of macrophages (Figure 4F). Differentially expressed genes (DEGs) were subsequently extracted from the macrophage-enriched groups in both datasets for downstream functional enrichment and pathway analysis.

### Myocardial infarction cell model induction

To simulate myocardial infarction (MI) conditions, neonatal rat cardiomyocytes were first cultured under normoxic conditions for 48 h to allow cell attachment and stabilization. After this pre-incubation period, the cells were washed twice with PBS to remove the regular culture medium and then transferred to serum-free, low-glucose DMEM. Subsequently, the cells were placed in an anaerobic culture chamber (C31, MGC AnaeroPack, Mitsubishi) and sealed with a CO_2_ gas-generating bag (A07, MGC AnaeroPack, Mitsubishi) to maintain an oxygen concentration below 1% at 37 °C for 6 h to induce oxygen-glucose deprivation (OGD). Following the hypoxic treatment, the cells were harvested for subsequent experiments. All animal procedures were approved by the Animal Ethics Committee of Guizhou Medical University (approval number: 2503312).

### Cell culture conditions

All cells were cultured in DMEM containing 10% fetal bovine serum (FBS) and 1% penicillin-streptomycin under 37 °C and 5% CO_2_ conditions. NO levels were assessed using a Griess-based biochemical kit.

### Wound healing assay (scratch assay)

After 24 h of co-culture, a scratch wound was made on the cardiomyocyte monolayer using a sterile pipette tip. Cells were then cultured in fresh medium for 24 h, and the wound closure was observed at 0 and 24 h using a phase-contrast microscope. Images were captured to assess the extent of wound healing, with the closure percentage calculated as follows: The experimental groups included: Blank control group (no treatment), Quercetin group (pretreated with quercetin). Cardiomyocytes were pretreated with quercetin (final concentration: 20 μM) for 24 h prior to injury induction. Quercetin was dissolved in DMSO and diluted in the culture medium, maintaining a final DMSO concentration below 0.1% (v/v). A vehicle control group (0.1% DMSO without quercetin) was included for comparison.

### qPCR analysis

Cardiomyocytes were treated with 20 μM quercetin or vehicle control (0.1% DMSO) for 24 h before RNA extraction. After co-culture, the cardiomyocytes were collected. Total RNA from rat cardiomyocytes was extracted using TRIzol and reverse transcribed into cDNA. qPCR was performed using SYBR Green or TaqMan probes to measure the expression of vegfa, ggt1, ptk2. Data were analyzed using the 2^-ΔΔCt method, with GAPDH as reference genes. The reaction primers used for key genes are as follows:

**Table:** 

Primer	Sequence (5’–3’)
F- Ggt1	AGAGACGGTGACTATGCCGAAG
R- Ggt1	GGTCCTCAACCGTCATAATGCC
F- Vegfa	CTGCTGTAACGATGAAGCCCTG
R- Vegfa	GCTGTAGGAAGCTCATCTCTCC
F- Ptk2	ACATCAAGGCGTGTACCTGAGC
R- Ptk2	GTGAGGATGGTCAAACTGACGC

### Molecular docking analysis

The 2D structures of the core active components were downloaded from the PubChem database (https://pubchem.ncbi.nlm.nih.gov/) and converted into 3D structures using ChemBioOffice Ultra 13.0.2-Chem3D. Energy minimization was performed to optimize these structures. The 3D structures of the core target proteins were retrieved from the Protein Data Bank (PDB) (https://www.rcsb.org/) and preprocessed using PyMOL 2.5.2 to remove water molecules and irrelevant residues. The docking grid box was defined with AutoDockTools-1.5.7 to encompass the entire protein structure. Semi-flexible molecular docking was carried out using AutoDock Vina (version 1.1.2), and the binding affinity (expressed as absolute binding energy) was calculated. Higher absolute binding energy values corresponded to stronger receptor-ligand interactions. The docking results were visualized and analyzed using PyMOL and Discovery Studio (version 4.5).

### Western blot analysis after Quercetin treatment

To assess the time-dependent effects of Quercetin on the expression of core genes, neonatal rat cardiomyocytes were treated with 20 μM Quercetin for 0, 12, 24, 36, and 48 h following oxygen-glucose deprivation (OGD) induction. At each time point, cells were harvested and lysed in RIPA buffer (Beyotime, Shanghai, China) containing protease and phosphatase inhibitors. Protein concentrations were measured using the BCA assay (Thermo Fisher Scientific).

Equal amounts of protein (30 μg per lane) were separated by 10% SDS-PAGE and transferred to PVDF membranes (Millipore, United States of America). The membranes were blocked with 5% non-fat milk for 1 h at room temperature and then incubated overnight at 4 °C with the following primary antibodies: anti-GGT1 (1:1000, Abcam, ab55138), anti-PTK2 (1:1000, CST, #3285), anti-VEGFA (1:1000, Abcam, ab52917), and anti-GAPDH (1:5000, Proteintech, 60004-1-Ig) as a loading control. After washing, membranes were incubated with HRP-conjugated secondary antibodies (1:5000, CST, #7074) for 1 h at room temperature. Protein bands were visualized using an ECL detection system (Bio-Rad, United States of America), and signal intensity was quantified using ImageJ software. All experiments were repeated three times independently.

### Statistical analysis

Survival analysis was performed using the survival package in R. Log-rank tests were applied to assess survival differences between groups. The wilcox test was used to compare differences between two or more groups, and Wilcoxon tests were used for pairwise comparisons. Spearman correlation analysis was used to determine the relationship between variables. All statistical analyses were performed using R versions 4.1.0 and 4.0.0, and a p-value <0.05 was considered statistically significant.

## Result

### Identification and enrichment analysis of key compounds from Peony and Licorice: potential therapeutic implications

**FIGURE 1:**
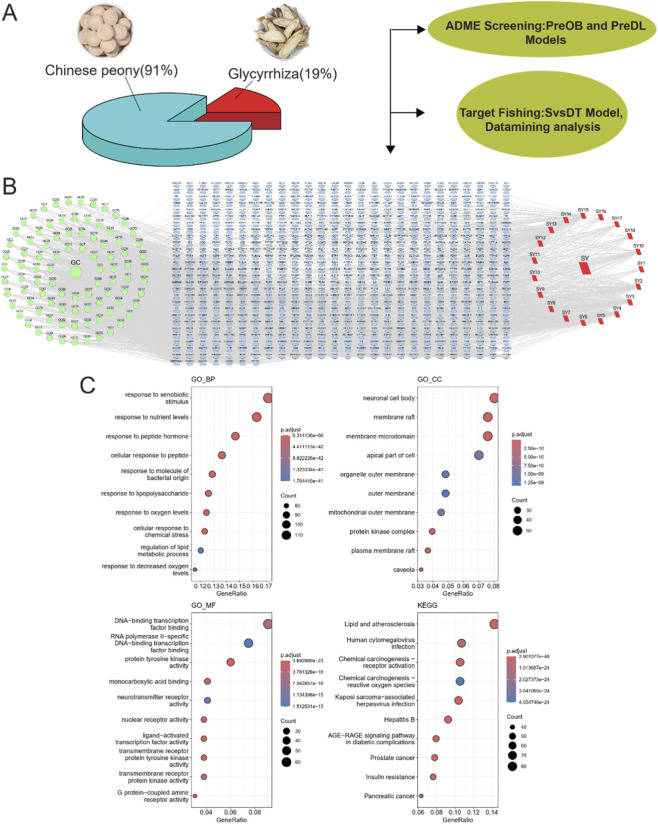
Analysis of active compounds and therapeutic potential of Peony and Licorice. **(A)** Pie chart displaying the composition of active compounds from Peony and Licorice. **(B)** Component-target network constructed, the network contains 821 nodes and 3,059 edges. **(C)** GO and KEGG enrichment analysis for the key targets of Peony and Licorice compounds.

We explored the therapeutic potential of a combination of two commonly used natural drugs, Peony and Licorice, by analyzing their active compounds and corresponding targets. Peonycompounds were screened using the TCMID database and further evaluated using the Swiss ADME software based on pharmacokinetic properties such as ADME. We identified a total of 110 effective compounds from the combination of Peony and Licorice, including 91 compounds from Peony and 19 compounds from Licorice (Supplementary Table S1). The resulting component-target network, constructed in Cytoscape, contained 821 nodes and 3,059 edges (Figures 1A,B). The core active compound identified was Quercetin (Table 1).

**TABLE 1:** Top 20 components of the network.

Number	Degree
GC88	143
SY3	138
SY13	118
SY2	104
SY15	101
GC90	101
SY4	98
SY7	90
SY6	68
SY19	64
GC13	61
SY8	52
GC10	44
SY11	41
SY10	39
GC7	38
GC11	38
GC14	36
GC6	35
SY5	33

GO enrichment analysis revealed that the key targets of these compounds were significantly enriched in biological processes such as response to peptide hormone, response to nutrient levels, and response to xenobiotic stimulus, as well as cellular components like membrane raft and neuronal cell body. Molecular functions such as DNA-binding transcription factor binding and pathways like chemical carcinogenesis - receptor activation and human cytomegalovirus infection were also significantly enriched. KEGG pathway analysis further highlighted the involvement of these compounds in pathways such as Lipid and Atherosclerosis and Human cytomegalovirus infection, which are critical to metabolic regulation and immune responses (Figure 1C). These findings suggest that Quercetin and other key compounds may have therapeutic potential in targeting inflammatory and metabolic disorders.

### Mendelian randomization

**FIGURE 2:**
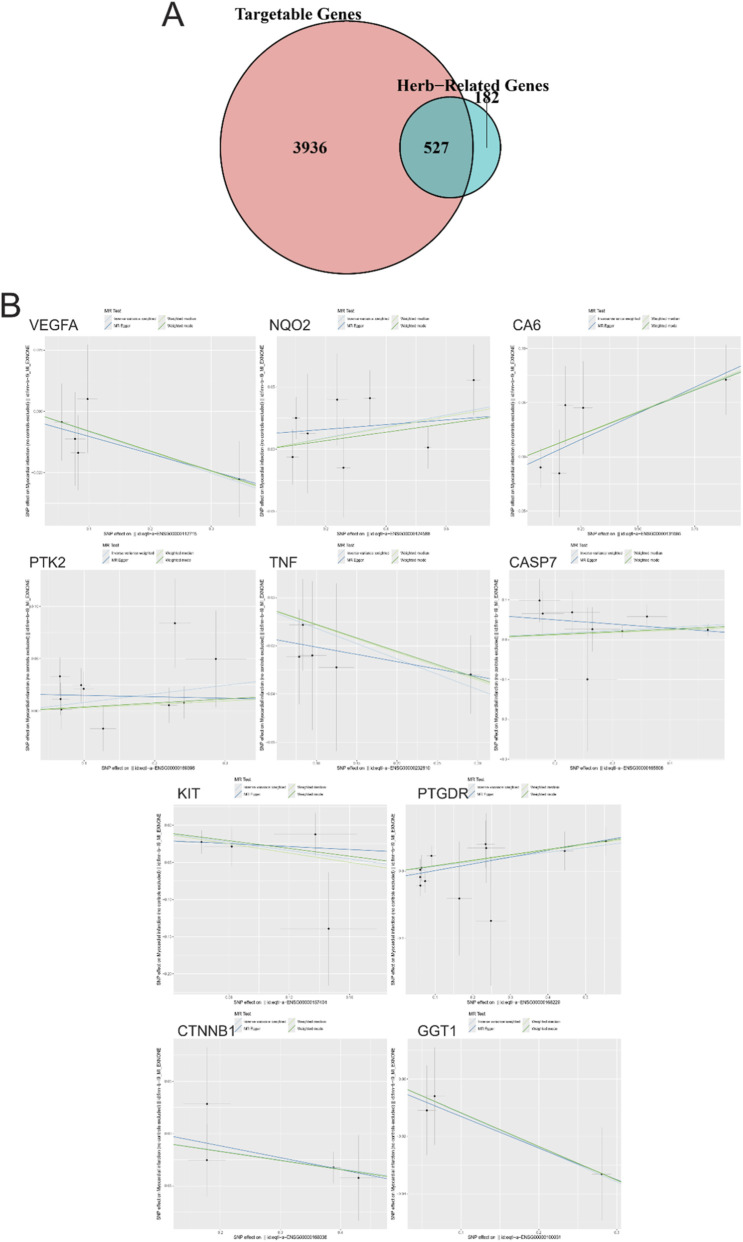
Intersection of druggable target genes and Mendelian randomization (MR) analysis results for myocardial infarction (MI). **(A)** Venn diagram showing the intersection of target genes from Peony and Licorice natural compounds with a public druggable target gene set. **(B)** Mendelian Randomization (MR) analysis results for the identified 10 genes with causal relationships to eQTL-positive outcomes in myocardial infarction-related samples.

We intersected a public druggable target gene set with the target genes of Peony and Licorice natural compounds, resulting in 527 genes. A Venn diagram of these intersected genes is shown in Figure 2A. Using summary statistics from myocardial infarction-related samples, we identified the outcome ID: finn-b-I9_MI_EXNONE. Using the extract_instruments and extract_outcome_data functions, we extracted causal relationships related to the outcome.

**FIGURE 3:**
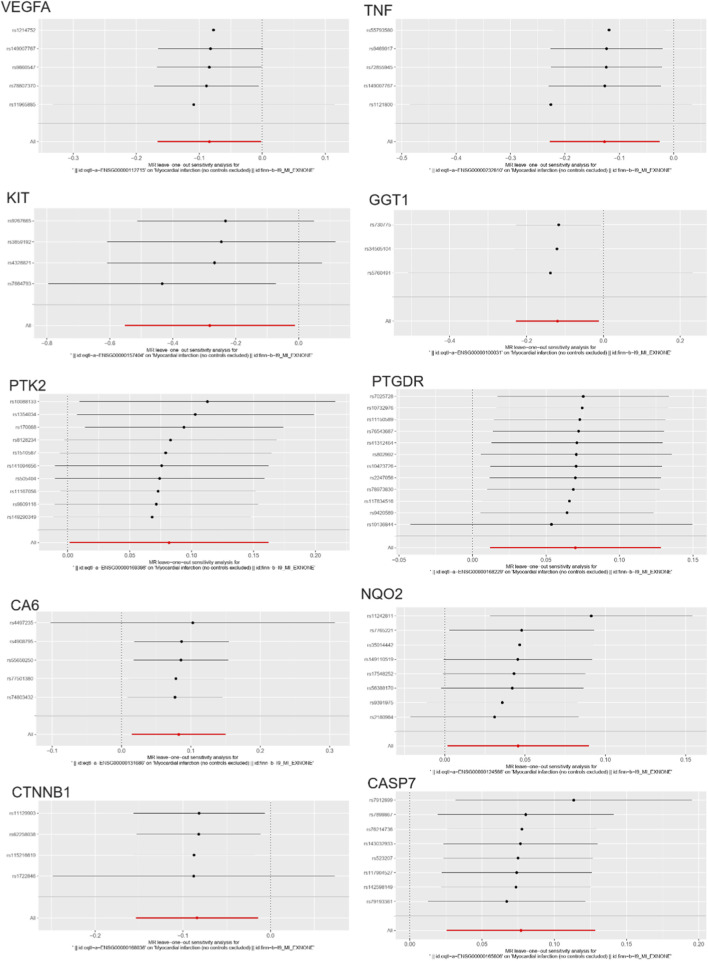
Sensitivity analysis of causal relationships identified through Mendelian randomization. Forest plot showing the sensitivity analysis of the 10 identified causal relationships.

Through Mendelian Randomization (MR) analysis, we identified 10 pairs of module genes with causal relationships to eQTL-positive outcomes (Figure 2B, IVW p-value <0.05). The corresponding genes included VEGFA, NQO2, CA6, KIT, PTGDR, PTK2, TNF, CASP7, CTNNB1, and GGT1. The causal relationships revealed that NQO2 (1.047; 1.002–1.094; P = 0.042), CA6 (1.886; 1.015–1.162; P = 0.017), PTGDR (1.073; 1.012–1.137; P = 0.018), PTK2 (1.085; 1.002–1.176; P = 0.046), and CASP7 (1.080; 1.026–1.137; P = 0.003) were associated with high risk of myocardial infarction. In contrast, VEGFA (0.919; 0.847–0.998; P = 0.044), KIT (0.754; 0.575–0.988; P = 0.041), TNF (0.881; 0.796–0.974; P = 0.014), CTNNB1 (0.920; 0.858–0.986; P = 0.018), and GGT1 (0.887; 0.796–0.989; P = 0.030) were associated with lower risk of myocardial infarction. To assess the reliability of these causal relationships, we performed a sensitivity analysis. The results indicated that excluding any single SNP did not significantly affect the overall error line, confirming the robustness of the 10 identified causal relationships (Figure 3).

### Heterogeneity and colocalization analysis of causal relationships in myocardial infarction-related genes

**FIGURE 4:**
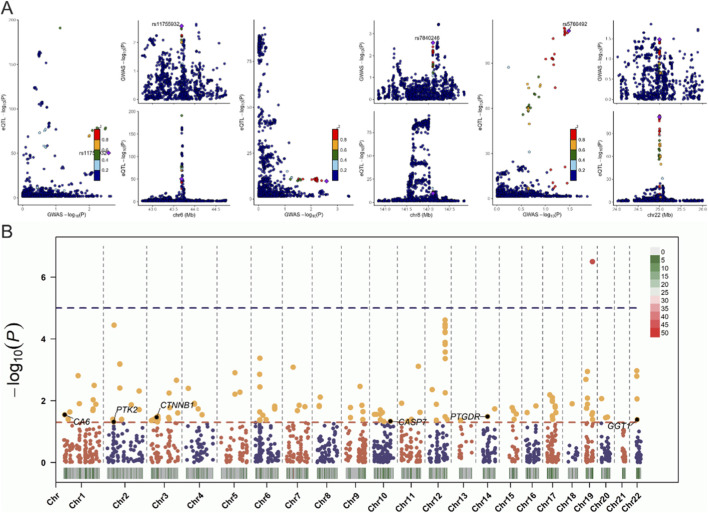
Colocalization and heterogeneity analysis for causal relationships in myocardial infarction (MI). **(A)** Colocalization analysis (coloc) showing the SNP.PP.H4 values for 10 genes, with GGT1, PTK2, and VEGFA demonstrating strong colocalization (SNP.PP.H4 > 0.75), indicating a significant overlap between eQTL and GWAS signals. **(B)** Manhattan plot illustrating the colocalization analysis for the 10 identified causal genes, highlighting the significant overlap in genetic signals for GGT1, PTK2, and VEGFA.

We conducted a heterogeneity analysis on the 10 identified causal relationships, and the results showed that all passed the heterogeneity test (Supplementary Table S2). Additionally, we performed a colocalization (coloc) analysis at the eQTL-GWAS level for these 10 genes. The GGT1, PTK2, and VEGFA genes showed colocalization SNP.PP.H4 values greater than 0.75 (Figures 4A,B), indicating that these genes have a strong overlap between their expression quantitative trait loci (eQTL) and genome-wide association study (GWAS) signals. This suggests a potential shared genetic basis for the identified causal relationships, further supporting their relevance in the context of myocardial infarction.

### Single-cell analysis of core genes related to myocardial infarction

**FIGURE 5:**
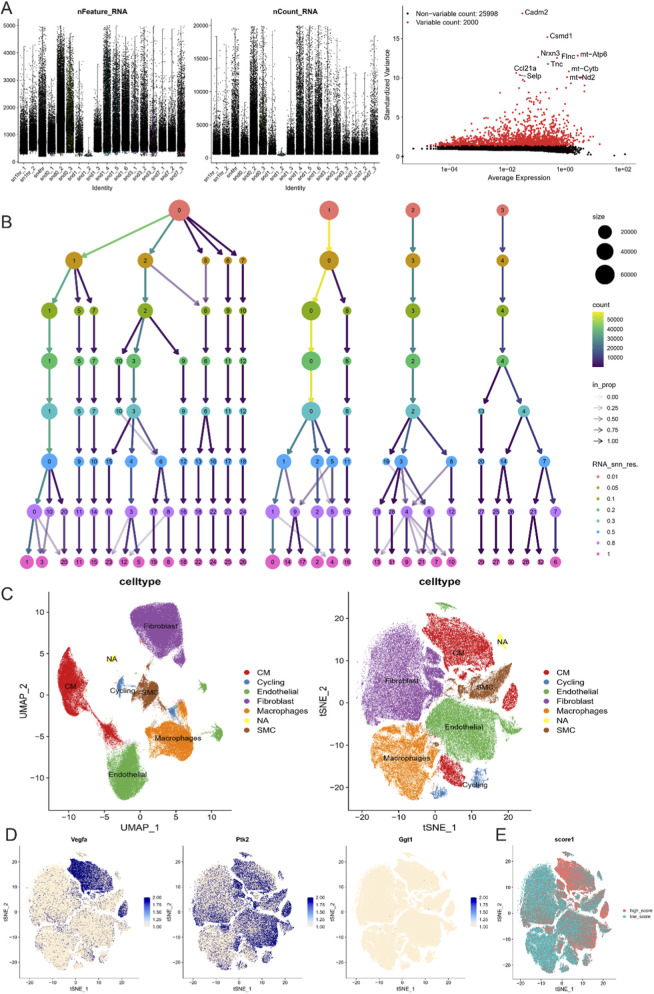
Single-cell RNA sequencing analysis of myocardial infarction samples. **(A)** Violin plots showing the distribution of feature RNA count and total RNA count, with a volcano plot for identifying high-variance genes. **(B)** Hierarchical clustering of cells at different resolutions, showing the identification of various cell subpopulations, including CM, Cycling, Endothelial, Fibroblast, Macrophage, SMC, and NA. **(C)** UMAP and tSNE plots visualizing the distribution of different cell types, highlighting the separation of subpopulations. **(D)** Feature plots for the expression of three key genes (VEGFA, PTK2, GGT1) across different cell types. **(E)** UMAP scoring plot based on the AUCELL algorithm, showing gene set scores for the three key genes across different cell types.

We analyzed the single-cell RNA sequencing data from myocardial infarction samples, performing quality control as shown in Figure 5A. Using an appropriate resolution, we clustered the cells and identified several subpopulations, including CM, Cycling, Endothelial, Fibroblast, Macrophage, SMC, and NA (Figure 5B). These subpopulations were visualized using UMAP and tSNE (Figure 5C). Next, we examined the expression patterns of the three key genes identified earlier. VEGFA was predominantly expressed in CM cells, while PTK2 showed a more widespread distribution, and GGT1 did not exhibit a specific expression pattern (Figure 5D). Using AUCELL, we scored each cell based on the gene set composed of these three genes, finding that these markers effectively distinguished the CM subpopulation. The CM cells exhibited significantly higher scores for the gene set, further supporting their role in myocardial infarction-related pathways (Figure 5E). These findings suggest that VEGFA plays a central role in the CM cell subpopulation, which is critical for heart muscle function and repair following myocardial injury. The broader distribution of PTK2 may indicate its involvement in various cell types related to tissue remodeling and repair, while the lack of specificity for GGT1 suggests its role may not be confined to a particular cell type but potentially involved in broader metabolic or inflammatory processes. This further underscores the importance of these key genes in the pathophysiology of myocardial infarction and heart failure.

### Quercetin modulates core gene expression and promotes myocardial cell recovery in myocardial infarction model

**FIGURE 6:**
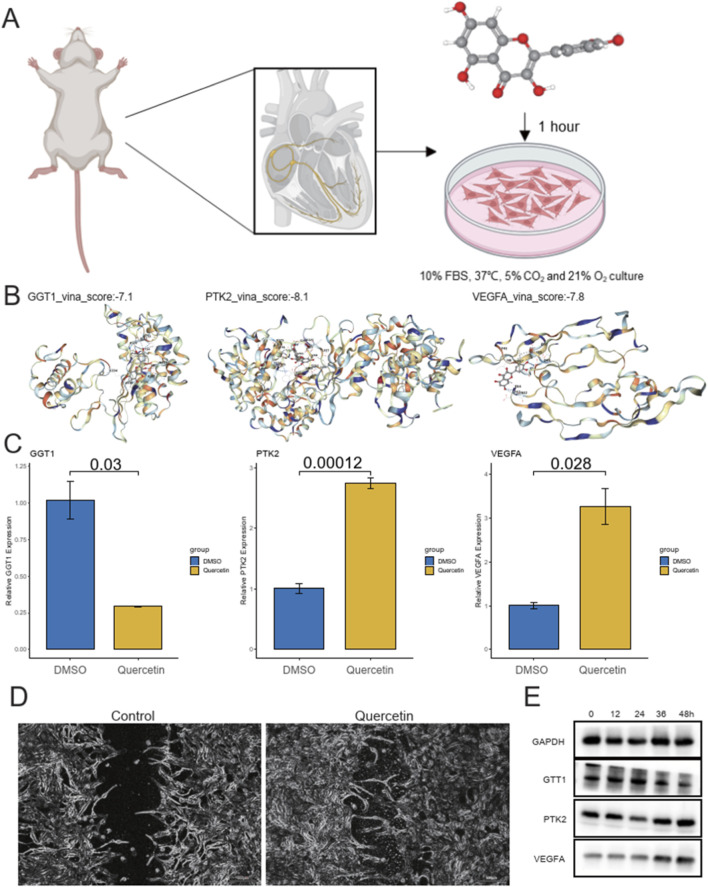
Experimental design, molecular docking, gene expression, and cell recovery in myocardial infarction model. **(A)** Schematic of the experimental procedure to simulate myocardial infarction (MI) in neonatal rat cardiomyocytes through oxygen-glucose deprivation (OGD) for 6 h, followed by hypoxic treatment under anaerobic conditions. **(B)** Molecular docking results showing the stable binding of Quercetin with three core genes (GGT1, PTK2, VEGFA) with binding energies: GGT1 (−7.1 kcal/mol), PTK2 (−8.1 kcal/mol), and VEGFA (−7.8 kcal/mol). **(C)** Bar plots showing the relative gene expression of GGT1, PTK2, and VEGFA in response to Quercetin treatment compared to DMSO controls. **(D)** Wound healing images showing the enhanced rate of myocardial cell recovery and growth in Quercetin-treated cells compared to controls. **(E)** Western blot validation of protein expression changes in GGT1, PTK2, and VEGFA following Quercetin treatment in cardiomyocytes.

To simulate myocardial infarction (MI) conditions, neonatal rat cardiomyocytes were first cultured for 48 h under normoxic conditions and then subjected to oxygen-glucose deprivation (OGD) for 6 h (Figure 6A). After washing the cells to remove the regular culture medium, they were cultured in serum-free low-glucose DMEM under anaerobic conditions to maintain an oxygen concentration of less than 1% for 6 h. Following hypoxic treatment, the cells were harvested for further experiments. We then performed molecular docking to assess the interaction between Quercetin and three core genes, GGT1, PTK2, and VEGFA. The docking results indicated that the proteins of these genes stably bound with Quercetin, showing stable binding energies (Figure 6B). Upon adding Quercetin, the expression levels of these three genes were significantly altered, demonstrating the drug’s influence on gene expression (Figure 6C). Additionally, scratch assays showed that Quercetin treatment enhanced the rate of myocardial cell recovery and promoted cell growth (Figure 6D). These results suggest that Quercetin could be a promising candidate for modulating gene expression and improving myocardial cell function in the context of myocardial infarction. As shown in Figure 6E, the protein levels of GGT1 and PTK2 gradually decreased following OGD exposure, whereas VEGFA expression increased in a time-dependent manner, reaching its peak at 36 h before slightly declining at 48 h. These dynamic changes are consistent with the qPCR results (Figure 6C), further confirming that Quercetin modulates the expression of these core genes at both the transcriptional and translational levels during myocardial injury and repair.

## Discussion

Cardiovascular diseases, particularly myocardial infarction (MI), remain a major global health burden, with limited effective treatments targeting the underlying mechanisms of the disease. Despite significant advances in pharmacological therapies, such as antiplatelet agents and beta-blockers, the long-term management of MI patients remains a challenge (Dauerman and Ibanez, 2021; Rao et al., 2024). Traditional Chinese medicine (TCM), with its long history of therapeutic applications, has garnered increasing attention as a complementary approach to managing cardiovascular conditions (Zhai et al., 2025). Among the many herbs used in TCM, Peony (Paeonia lactiflora) and Licorice (Glycyrrhiza uralensis) have demonstrated various pharmacological effects, including anti-inflammatory, antioxidant, and cardioprotective properties (Wang et al., 2025). However, their combined therapeutic potential in MI has not been thoroughly explored. This study aims to fill this gap by investigating the active compounds from Peony and Licorice and evaluating their potential therapeutic roles in MI through network pharmacology, Mendelian randomization (MR) analysis, and molecular docking.

Our study identified 110 active compounds from Peony and Licorice, with Peony contributing the majority of the compounds. The component–target network, composed of 821 nodes and 3,059 edges, highlighted the complex interactions between these compounds and their respective molecular targets. Among the key compounds, Quercetin emerged as a particularly promising candidate due to its strong therapeutic potential (Dehghani et al., 2023; Farazi et al., 2024; Albadrani et al., 2020). Gene Ontology (GO) enrichment analysis revealed that the targets of these compounds are involved in critical biological processes, such as responses to peptide hormones and xenobiotic stimuli, and pathways related to lipid metabolism and atherosclerosis. These findings suggest that Peony and Licorice compounds, particularly Quercetin, may exert their therapeutic effects by modulating inflammation, metabolic pathways, and cardiovascular homeostasis, which are key factors in the pathogenesis of MI.

Mendelian randomization (MR) analysis further identified ten genes associated with MI risk, including VEGFA, NQO2, PTK2, and TNF. Among these, VEGFA and TNF, which have been implicated in myocardial damage and tissue repair, are critical regulators of cardiac function following ischemic injury. The MR analysis revealed causal relationships between these genes and MI, indicating that Quercetin and other active compounds from Peony and Licorice may modulate these genes to reduce the risk of MI. This is consistent with existing studies that have highlighted the roles of these genes in regulating inflammation, angiogenesis, and tissue remodeling in cardiovascular diseases. Our findings align with previous literature reporting the cardioprotective effects of Quercetin, particularly its ability to reduce oxidative stress and inflammation—two central processes in MI progression. Previous studies have shown that Quercetin can promote angiogenesis through VEGFA activation and improve myocardial recovery following ischemic events (Dehghani et al., 2021; Luo et al., 2023). Similarly, Licorice compounds such as glycyrrhizin have demonstrated anti-inflammatory and antioxidant effects that are beneficial in preventing myocardial injury (Zu et al., 2021; Yu et al., 2024). These studies corroborate our findings and provide further evidence for the therapeutic potential of Peony and Licorice in MI management.

Recent evidence has further revealed that membrane lipid rafts serve as critical signaling platforms regulating inflammation and oxidative stress, and that dysregulation of raft-associated signaling complexes contributes to endothelial activation and vascular injury (Radenkovic et al., 2020). Given that Quercetin, identified in our study as the major active flavonoid derived from the combined Peony and Licorice components, possesses well-documented anti-inflammatory and antioxidant properties, it is plausible that part of its cardioprotective efficacy in MI may converge through the modulation of lipid raft–dependent signaling pathways. By stabilizing or reorganizing these membrane microdomains, Quercetin could attenuate the assembly of pro-inflammatory receptor complexes and suppress redox-sensitive signaling cascades, ultimately reducing myocardial inflammation and oxidative damage. This hypothesis provides an additional mechanistic framework linking our findings to emerging molecular models of cardiovascular inflammation.

However, there are several limitations to our study. While network pharmacology and MR analysis provide valuable insights, the computational nature of these approaches limits their ability to capture the full complexity of biological interactions *in vivo* (Sun et al., 2024). Although we identified key genes associated with MI and validated their expression through *in vitro* assays, the lack of *in vivo* validation remains a primary limitation of the present study. *In vitro* experiments, while useful for mechanistic exploration, cannot fully replicate the complex physiological and pathological environment of myocardial infarction (Chan et al., 2020). Future *in vivo* studies using myocardial infarction animal models are therefore essential to confirm whether Quercetin truly exerts cardioprotective effects by modulating VEGFA, PTK2, and GGT1, as predicted in our bioinformatic and molecular docking analyses. Such experiments would also help elucidate the pharmacokinetics, biodistribution, and dose–response relationships of Quercetin, thereby strengthening the translational potential of these findings.

Additionally, the multi-component nature of TCM formulations makes it challenging to isolate the specific contributions of individual compounds. More detailed studies are required to dissect the synergistic effects among the active ingredients and to clarify their molecular mechanisms in MI. Another limitation lies in the lack of large-scale clinical trials to validate the therapeutic efficacy of Peony and Licorice compounds in MI patients (Wereski et al., 2022; Zhang et al., 2024). While our results are promising, future clinical studies are needed to evaluate the safety, optimal dosage, and long-term outcomes of these compounds in MI treatment. The therapeutic potential of Quercetin and other identified compounds should be explored as adjunctive therapies alongside conventional treatments to determine their efficacy and safety in real-world clinical settings.

Building upon the findings of this study, several avenues for future research can be envisioned. First, *in vivo* validation using animal models of myocardial infarction is crucial to confirm the cardioprotective mechanisms of Quercetin observed *in vitro*. Such studies would allow the evaluation of myocardial repair, fibrosis reduction, and angiogenesis in a physiological context. Second, multi-omics approaches, including transcriptomics, proteomics, and metabolomics, could provide deeper insights into the molecular networks modulated by Peony- and Licorice-derived compounds. These integrated analyses may uncover additional therapeutic targets or biomarkers for disease monitoring. Third, future investigations should explore formulation optimization and pharmacokinetics of Quercetin to improve its bioavailability and stability, thereby enhancing its translational potential. Finally, early-phase clinical trials will be essential to determine the safety, tolerability, and preliminary efficacy of Quercetin-based therapies in patients with MI. Such studies will bridge the gap between computational and preclinical research and pave the way for evidence-based integration of traditional Chinese medicine into modern cardiovascular therapeutics.

## Data Availability

The datasets presented in this study can be found in online repositories. The names of the repository/repositories and accession number(s) can be found in the article/Supplementary Material.
